# Extraction and reduction of parameters from hand-drawn Archimedes spirals for clinical tremor assessment

**DOI:** 10.1016/j.heliyon.2024.e34911

**Published:** 2024-07-20

**Authors:** Furrukh Khan, Jessie Xiaoxi, Andrew Uehlin, Brian Dalm, Evan Thomas

**Affiliations:** a Department of Electrical and Computer Engineering, The Ohio State University, Columbus, OH, USA; b Image Guided Therapy Devices, Philips, CO, USA; c Department of Neurosurgery, The Ohio State University, Columbus, OH, USA; d Department of Radiation Oncology, The Ohio State University, Columbus, OH, USA

**Keywords:** Tremor assessment, Tremor quantification, Archimedes spiral, Tremor feature reduction

## Abstract

**Background:**

Patients’ hand-drawn Archimedes spirals are widely used in the neurological community to grade tremors. These spirals are either drawn on paper and Xeroxed/scanned into digital images or digitizing tablets are used for the drawings. This process introduces artifacts such as variable widths of the drawn lines with varying pixel grey scale values. Xeroxing introduces additional artifacts resulting from paper misalignments. These artifacts and the presence of the reference spiral in the image complicate an automatic extraction of a mathematical spiral signal from the image.

**New methods:**

We introduce a mathematical mapping that transforms the image pixels of the patient's hand-drawn spiral into a *one-dimensional* discrete signal that can be used for mathematical analysis.

**Results:**

A cohort of 18 hand-drawn spirals with various artifacts is used to validate our method.

We extract the parameters of the discrete signals and show that the signals can be represented by truncating to as few as 150 parameters with a truncation RMS error of 6.26 % across the cohort. Using only 150 features makes machine learning a viable option for future applications. Furthermore, our method can be used to evaluate the frequency and the amplitude of the tremor.

**Comparison with existing methods:**

In existing methods, the patient draws the spiral on a digitizing tablet, and features are extracted from this data for machine learning. We recognize that a vast majority of hospitals are still using the pencil-and-paper approach, and there is an abundance of ready-to-be-mined tremor-related data already stored as paper or digitized drawings. Our procedure is equally applicable to Xeroxed documents as well as files generated from digital tablets.

**Conclusions:**

We have validated a new procedure requiring minimal user intervention to automatically extract a patient's hand-drawn spiral as a discrete mathematical *one-dimensional* signal from a scanned image or a file from a digital tablet.

## 1 Introduction

**Fig. 1:**
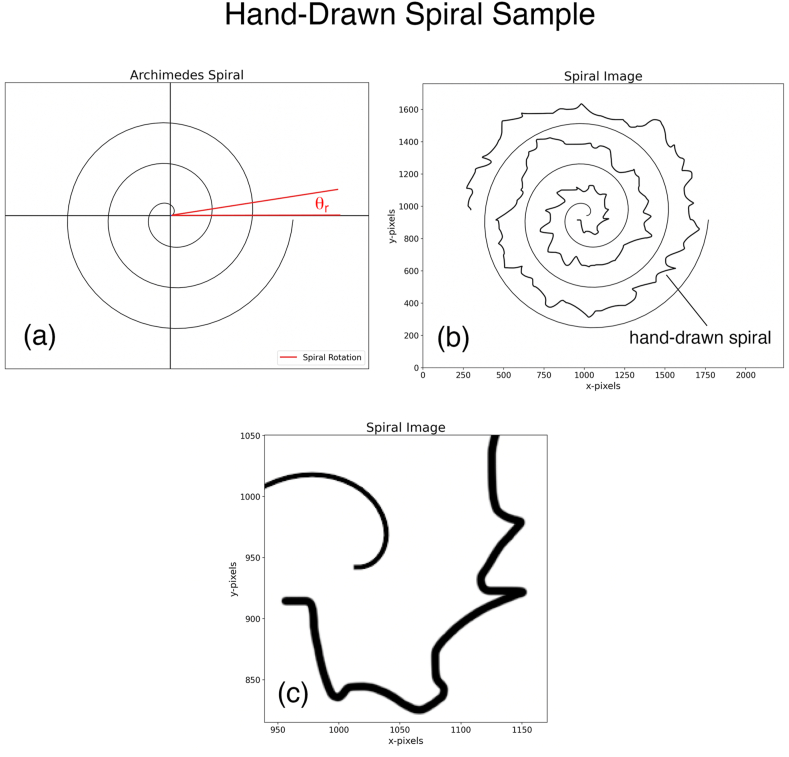
(a) Archimedes Spiral rotated by a positive angle $\theta_{r}$**(b)** Image in *Spiral Space* showing the spiral, as well as the hand-drawn spiral. **(c)** The magnified portion of the image in (b) showing that the spiral and the patient's hand-drawn spiral, have multi-pixel widths.

The Discovery of the Archimedes spiral [1] (Fig. 1a) in the 3rd century BC is attributed to Canon of Samos [2], a disciple of Archimedes [3]. This spiral is extensively used in medicine to study various forms of tremors [4]. A patient is asked to draw a spiral by hand, on paper or a digitizing tablet, inside the space of an Archimedes spiral, and a physician grades the severity of the tremor based on the patient's drawing [[5], [6], [7], [8]]. An example is shown in Fig. 1b where we have drawn the spiral ourselves to mimic a patient's hand-drawn spiral. It is extremely advantageous to be able to convert the patient's spiral into a *one-dimensional* function (signal) so that mathematics can be used to process the signal, e.g., determining the tremor amplitude, analyzing the spectrum of the signal to determine the tremor frequency, applying statistics to make inferences, or applying machine learning techniques to make predictions.

For a review of the laboratory tests used for tremor diagnosis and classification, including the Archimedes spirals, the reader is directed to a recent review by Angelini et al. [9] The spirals are often drawn on paper by a patient using a pen or a pencil, or on a tablet using a digitizing pen. To convert the paper-drawings to digital images, the document is Xeroxed and scanned, resulting in the introduction of image artifacts. One outcome is the presence of regions of varying light-grey pixels in the background instead of pure white pixels in the scanned document. This problem can often be fixed by simply altering the contrast of the image. More serious obstacles to extracting the signal are that the documents may not be precisely aligned with the beds of the Xerox machines/scanners, which causes the spirals to get rotated and translated by unpredictable amounts. Furthermore, the drawn lines in Xeroxed documents as well as in digital tablet drawings are not single-pixel wide, instead, they have variable multi-pixel widths (Fig. 1c) and are also composed of different grey scale values. There may also be other artifacts such as the drawings crossing the target Archimedes spiral, or having gaps and loops. These artifacts, along with the presence of the target/reference spiral in the image, make it challenging to extract a clean, single-valued *one-dimensional* discrete signal from the image using image processing techniques.

To overcome these challenges there has been work in the literature to circumvent the paper-drawn technique and instead use digitizing tablets [[10], [11], [12], [13], [14], [15]]. The patient writes or draws the spiral on the tablet and the (x,y) pixel data from the digitizing pen is fed to a computing device, such as a microcontroller. The computing device extracts a feature set from the data, and statistical/machine learning algorithms are used to assess and quantify the tremor. We take a different approach in this paper. We realize that a vast majority of hospitals are still using the pencil-and-paper approach, and there is an abundance of ready-to-be-mined tremor-related data already stored as paper or digitized drawings. In this paper, we outline a procedure that is not based on image processing and is equally applicable to Xeroxed documents as well as files generated from digital-tablets. The procedure automatically extracts a clean signal from an image with minimal user interaction.

This paper aims to extract a one-dimensional discrete signal from an image corresponding to a spiral drawn by a patient on paper or a digitizing tablet. This image consists of pixels containing the hand-drawn spiral as well as the reference spiral. Our method which requires minimal user interaction, first utilizes the reference spiral pixels to determine the mathematical parameters of the reference spiral. These parameters are then used to determine a mapping that mathematically transforms all the pixels of the original image space into a new image space in such a way that (in the new space) the reference spiral pixels fall on a straight line. This mapping enables us to extract a one-dimensional mathematical signal of the patient's spiral from the transformed spiral pixels with minimal user interaction. This paper further aims to investigate the feasibility of using predictive Machine Learning algorithms that use the Fast Fourier Transform (FFT) [16] parameters of the spiral signals as input features. We aim to determine whether the FFT parameters can be truncated to an appropriately low number for Machine Learning to be a viable option. The accuracy of the truncation is tested by determining the error between the original signal and the inverse FFT [17] of the signal with truncated parameters.

## 2 Methods

### 2.1 Mathematical preliminaries

In this section, we cover a few mathematical details relevant to this paper.

#### 2.1.1 Basic equations in Spiral Space

We start with a 2D space with polar coordinates r∠θ. We name this space *Spiral Space*. An Archimedes Spiral in this space is defined as^1^,

r=bθ,

(1) r>0,chooseb>0⟹θ>0.

we choose b>0 in this paper which implies that θ>0. The spiral (1) can be rotated around its origin by replacing θ by $\theta +\theta_{r}$,

$$r=b\left(\theta +\theta_{r}\right)\text{,}$$

(2) $$r>0,\text{choose}\;b>0⟹-\theta_{r}<\theta <\infty \text{,}$$

where $\theta_{r}$ is the angle of rotation. To express this rotated spiral in rectangular coordinates centered at the origin,

x=rcosθ

(3) y=rsinθ,

we use (2) and (3) to get,

$$x=b\left(\theta +\theta_{r}\right)\cos\;\theta \text{,}$$

(4) $$y=b\left(\theta +\theta_{r}\right)\sin\;\theta ,-\theta_{r}<\theta <\infty \text{.}$$

Fig. 1a shows a spiral rotated by a positive angle, $\theta_{r}$.

#### 2.1.2 Spiral equation from two points

Since eq. (4) has two unknown parameters, b and $\theta_{r}$, the equation for a centered spiral can be completely determined if we know the polar coordinates of any two points, $r_{11}∠\theta_{11}$ and $r_{22}∠\theta_{22}$, lying on the spiral. Inserting the coordinates of these two points into equation (2) we get two equations in two unknowns. Solving these equations gives the unknowns $\theta_{r}$ and b,

(5) $$\theta_{r}=\frac{r_{22}\theta_{11}-r_{11}\theta_{22}}{r_{11}-r_{22}}$$

(6) $$b=\frac{r_{11}-r_{22}}{\theta_{11}-\theta_{22}}$$

#### 2.1.3 Flattened Space

Our aim is to map the *Spiral Space* containing the spiral expressed by (2) to a new space, which we call *Flattened Space*, in which the spiral is *unraveled* or flattened into a straight horizontal line. Such a mapping is given by,

$$x=\alpha \left(\theta +\theta_{r}\right)\text{,}$$

$$y=\beta \left(r-b\left(\theta +\theta_{r}\right)\right)\text{,}$$

(7) $$-\theta_{r}<\theta <\infty \text{.}$$

where α and β are arbitrary constants. $\theta_{r}$ and b are given by (5), (6). We can see by substituting (2) into (7), that we get y=0 for all values of x. Also, as θ changes, x changes linearly with it, proportional to the value of α. Hence, the spiral is mapped into the x-axis through this mapping. In this paper, we choose α=1 and β=1 without loss of generality.

### 2.2 Zones and Branch Cut in the Flattened Space

For this study, we are given an image in some format (jpg, png, tiff etc.) with pixels (greyscale or color) containing the spiral as well as the patient's hand-drawn spiral, Fig. 1b. A magnified portion of this image is shown in Fig. 1c. Note that the spiral and patient's hand-drawing are not single-pixel but multiple-pixel wide, with different greyscale or color values. We are not given the actual equation of the spiral contained in the image pixels. To flatten the patient's hand-drawn spiral by using (7), we need an equation for the spiral, i.e., the parameters b and $\theta_{r}$. Later in the paper, we show explicitly how our procedure allows us to determine these parameters from the image; however, here we assume that we have access to these parameters.

As we increase θ in (2), r increases to trace the spiral, with θ varying from $-\theta_{r}$ to ∞. It is useful to rewrite this equation by redefining θ in terms of a new variable $\theta^{\prime }$, which is restricted to the interval 0 to 2π,

$$\theta =\theta^{\prime }+2\pi n\text{,}$$

(8) $$0<\theta^{\prime }<2\pi ,n=1,2,3\cdots$$

The first part of the spiral near the origin is traced by $-\theta_{r}<\theta^{\prime }<2\pi$.

**Fig. 2:**
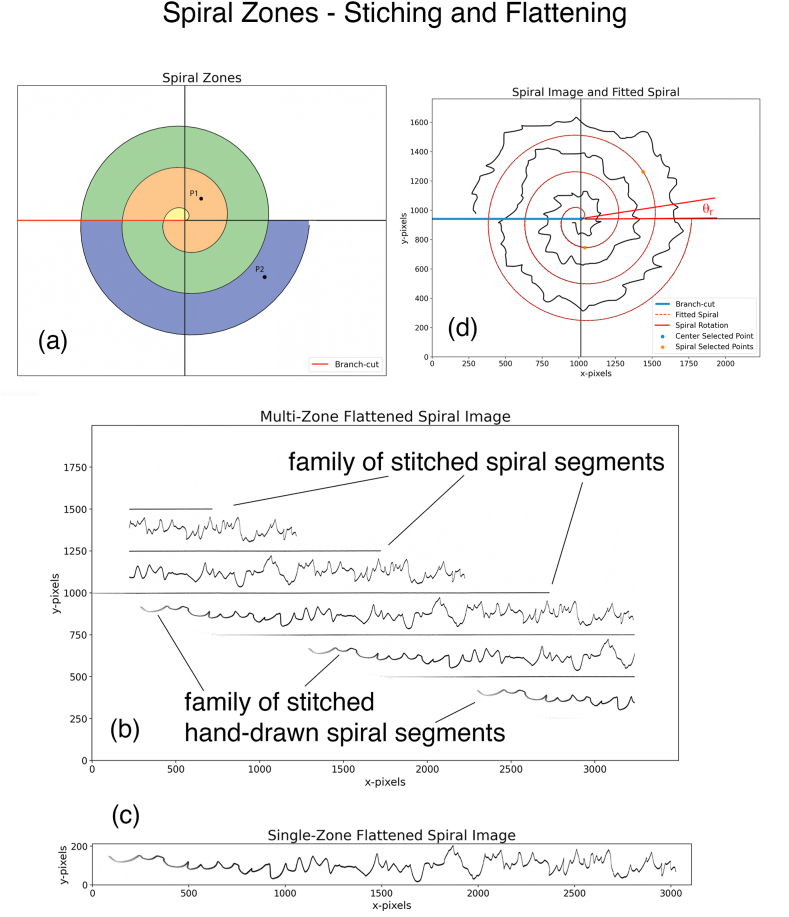
(a) Zones. P1, P2 belong to zones n=1, n=3. **(b)** Flattened spiral image of Fig. 1b **(c)** User-selected pixels of a single stitched hand-drawing from the image in Fig. 2b. **(d)***Spiral Space* of Fig. 1b and the fitted spiral.

To draw the spiral, we increase $\theta^{\prime }$, and every time it reaches 2π, i.e., the spiral crosses the negative *x-axis*, we increase n by 1 and reset $\theta^{\prime }$ to 0. We denote the negative *x-axis* as the *branch cut* in *Spiral Space*, as shown in Fig. 2a.

We use (7) and (8) with α=β=1 to map all the pixels in the image to the *Flattened Space*. Note that some of these pixels are from the spiral, some form the patient's hand-drawn spiral, while most others are just white pixels (or light grey pixel artifacts introduced by the Xerox machines/scanners). Our aim is to map each pixel of the whole image, not just the spiral pixels.

This brings us to the central problem in flattening the image: if we have a random point (pixel), r∠θ, in *Spiral Space*, how do we determine which integer n=1,2,3⋯ in (8) to assign to it so that we can use (7) and (8) to map it into *Flattened Space*? The image gives us no straightforward way to determine this number. If n is not determined, then any point P in the *Spiral Space* will map not only to one single point (x,y) in the *Flattened Space,* but to a family of points, each point being associated with a give n 1,2,3⋯,

(x,y)⟹(x+2πn,y+2πnb)

(9) n=1,2,3⋯

We can rephrase this problem in a different way by introducing the concept of zones. The set of points in *Spiral Space* that should be associated with n = 1 for the mapping is assigned zone1, all points which need n = 2 are grouped into zone2, etc. Fig. 2a shows some zones in *Spiral Space* in different colors. In this figure, point P1 belongs to zone1 with n=1, and P2 belongs to zone3 with n=3. Now we rephrase the problem posed in the previous paragraph: how do we determine to which zone a random point in *Spiral Space* belongs? It is relatively easy to visually inspect the image and assign a zone to a point, but there is no easy way to programmatically assign a random pixel from the picture to a zone. It is impractical to visually inspect and label each of the tens of thousands of pixels in the image. It is also challenging to devise image processing algorithms to automatically determine the zones, given the aforementioned artifacts present in the images.

We solve this problem by using a simple scheme with minimal user intervention. We map each point in the image to *Flattened Space* by using not just one correct value of n, but instead mapping that pixel to a family of points (9) in *Flattened Space* by using a list of values for n=1,2,3,4. As a result, the segments of the spiral corresponding to different values of n automatically get stitched together (without the user's intervention) in *Flattened Space,* into a family of horizontal line segments with vertical spacings of 2πb, according to (9). In the same way, segments of the patient's hand-drawn spiral in different zones automatically get stitched together into a family of parallel flattened hand-drawn spirals with vertical spacings of 2πb. Fig. 2b shows an example of an image in *Flattened Space* using our scheme. The figure shows the family of stitched spiral segments as horizontal lines, as well as the family of stitched patient's hand-drawn segments. Recall that *Flattened Space* is also an image containing image pixels. Our aim is to extract that part of the image which only contains the pixels of one stitched hand drawing from the family of many parallel hand drawings in the image. We allow the user to make this selection (crop) in our software, by interactively choosing a rectangle bounding the pixels of the desired hand-drawing. This selection is saved to a new image shown in Fig. 2c. Thus, this scheme allows us to solve the problem of multiple zones by employing minimal user intervention.

Note that as the patient draws the spiral from the origin, $\theta =-\theta_{r}$, we move along the spiral with a distance s. The incremental distance, ds, is related to the incremental angle, dθ, as ds=rdθ. Using (2) in this equation, making the substitution $\theta \to \theta -\theta_{r}$, and integrating w.r.t. θ gives,

(10) $$s=\frac{1}{2}b\theta^{2}$$

For the extracted discrete signal, we want the x-axis to be in terms of s, therefore after mapping with (7) we need to use (10) to transform the x-axis of the discrete signal to the distance, s, along the spiral.

### 2.3 Software preliminaries

Python v3.8.2 is used to implement all the procedures presented in this paper. Python is developed under OSI (Open-Source Initiative) approved license [18,19], making it freely useable and distributable, even for commercial use. Python's packages *matplotlib* and *scipy* are used for plotting and scientific routines. It should be easy to convert our code to MATLAB [20] since the Python packages used in this paper are similar to the corresponding implementations in MATLAB. All the spirals, data, user guide, and software developed for the project in this paper are available in the authors' GitHub repository [21].

## 3 Results

### 3.1 Obtaining the discrete signal from the spiral image

In this section, we outline the steps in our approach to map the *Spiral Image* into *Flattened Space* and then extract the *one-dimensional* spatial discrete signal [22] from the patient's hand-drawn spiral. We organize our approach into 5 steps. Our Python program is also arranged around these steps. Our program implements interactive plots which respond to the user's mouse clicks on the screen. We use the spiral image shown in Fig. 1b to serve as an example in the rest of this section.

Step 1-1 Read the Spiral Image File

Pixels from the image file which contains the spiral as well as the patient's hand-drawn spiral (Fig. 1b), are read. A file that contains a log of important events is also defined.

Step 1–2 Determine the Equation of the Spiral

First, the user clicks on the interactive plot to determine the origin of the spiral, and then the user clicks twice to select two points on the spiral. Section “Spiral equation from two points*”* is followed to determine the parameters $\theta_{r}$ and b. Our program assumes that the first user-selected point lies in zone one while the other belongs to zone two. These parameters give us the equation of the fitted spiral (4). Fig. 2d shows that the fitted spiral is an excellent fit for the original spiral in the *Spiral Space*. This fitted spiral is used next to flatten the Spiral Image.

Step 1–3 Flatten the Spiral Image

The section “Zones and Branch Cut in the Flattened Space” is followed, and the equation for the spiral is used to map the Spiral Image to *Flattened Space*. Fig. 2b shows the family of stitched spiral segments, as well as the family of stitched patient's hand-drawn segments. It takes approximately 1 min and 20 s on a year 2000 Mac Book Pro laptop to complete this step.

Step 1–4 Crop to get a Single Stitched Patient's Hand-Drawn Spiral

The user clicks twice on the interactive plot to select a cropping rectangle to extract the part that only contains the pixels of a single stitched patient's hand drawing, Fig. 2c.

Step 1–5 Derive and Save the Discrete Signal

**Fig. 3:**
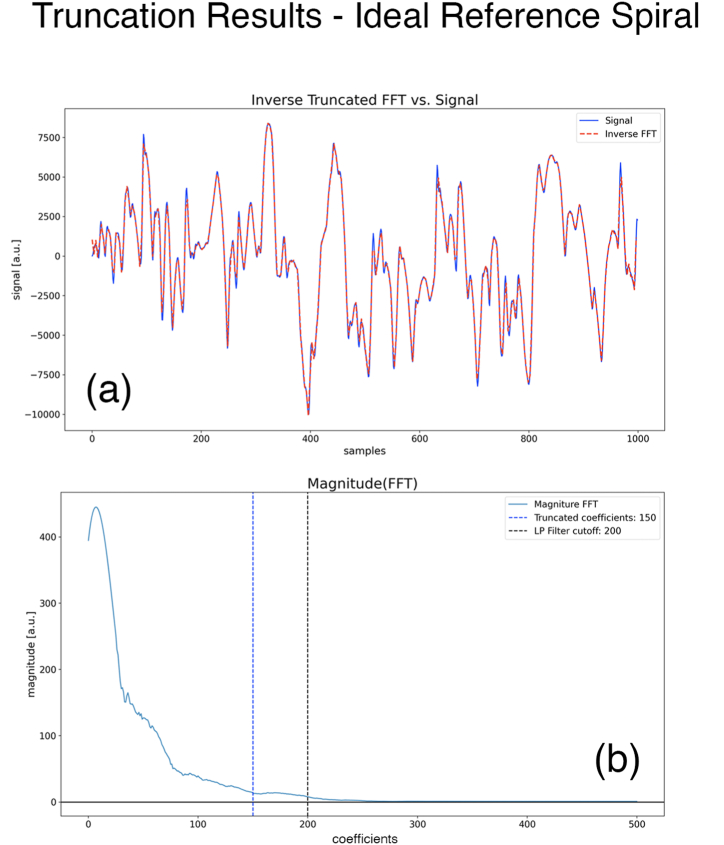
(a) Original normalized discrete signal extracted from the spiral in Fig. 1b, and the approximate discrete signal obtained from the FFT shown in (b) truncated to 150 coefficients. The RMS error between the two signals is 3.80 % **(b)** FFT of the discrete signal shown in (a).

The patient's hand-drawn spiral image from Steps 1–4 is multiple pixels wide with pixels having different greyscale (or color) values; it is not yet in the form of a discrete signal on which mathematical manipulations could be performed, such as determining its DFT [23] (Discrete Fourier Transform). A further complication is introduced by a scanned image having light grey (light color) pixels spread throughout the background pixels, which should ideally be white. The approach we take to extract a single-valued signal is to select each x-pixel (in the Python program) and search in the vertical direction to select the y-pixel having the highest greyscale (or color) value; we assign this y-pixel to the value of the signal corresponding to that x-pixel. A major benefit of this approach is that by choosing the highest greyscale (or color) value, the background light-grey pixels present in a scanned image are completely ignored if their strength is relatively light compared with the hand drawing. This technique works even better if the contrast of the original image is enhanced before processing. Now, we have a one-to-one correspondence between the x-values and the y-values, which constitutes a signal upon which mathematical manipulations can be performed. At this point, we have a signal with a large number of samples (pixels). For the spiral of Fig. 2, we end up with a number of x-pixels, $N_{original-pixels}=3228$. This number is too large to be a practical feature set for Machine Learning. We also need to correct the signal based on (10). This signal can potentially have some noise spikes in a very small region near the origin; in our program, the user is prompted to crop out this noise region. Finally, we resample the signal to N=1000 samples to reduce the number of parameters. This number is large enough to give us a Nyquist frequency which is much larger than the max tremor frequency (∼12 Hz) of interest for us. This is discussed in more detail in the discussion section below. Fig. 3a shows the signal obtained from the image, which has been normalized to have zero mean and its absolute value bounded by 1000 pixels. The program keeps track of how many pixels are being cropped in various steps throughout the procedure in a variable named cropping_ratio. The final cropped discrete signal with N=1000 samples is then converted to a (Python) Panda's data frame and saved as a csv (comma-separated value) file for the next step.

### 3.2 Obtaining the DFT (FFT) from the signal

We organize the task of analyzing the DFT [23] of the cropped discrete signal into 4 steps. Our interactive Python program which implements these steps is also structured around these steps. We continue to use the spiral image shown in Fig. 1b as a running example.

Step 2-1 Read the Discrete Signal from the csv File

The cropped discrete signal with N=1000 samples is read from the csv file and normalized. A file that contains the log of important events is created. The user also initializes the variable named cropping_ratio which was calculated in the steps above.

Step 2-2 Determine the FFT of the Discrete Signal

To determine the DFT, the popular and efficient algorithm, FFT, is employed. The FFT of a real-valued function is a complex number having a magnitude and a phase. For N sample points in the discrete signal, the number of coefficients, $N_{coeff}$, in the magnitude and phase FFT equals,

(11) $$N_{coeff}=\frac{N}{2}+1$$

Fig. 3a shows the original discrete signal while Fig. 3b shows its magnitude FFT. The x-axis consists of $N_{coeff}=501$ values, (11). A low pass filter is applied with a cutoff of ${0.4\times N}_{coeff}$ to eliminate high-frequency noise outside the band relevant to tremors. Determining tremor frequencies is discussed later in the discussion section. A Savitzky-Golay filter (window size:51, poly order:3)[24,25] is applied for the plots to better visualize the discrete spectrum; it is only applied for plotting and does not alter the data for subsequent calculations.

Step 2–3: Determine the Inverse FFT of the Truncated FFT

Fig. 3b ($N_{coeff}=501$), shows a rapid drop in the magnitude of the FFT with increasing coefficients. This observation leads us to assert that truncating the FFT at a relatively low number of coefficients, $N_{trunc}\ll N_{coeff}$, could result in a good approximation for the patient's hand-drawn spiral. We may be able to approximately represent the spiral signal with only ${2N}_{trunc}$ parameters, instead of the N samples required for the original discrete signal. The factor of 2 in ${2N}_{trunc}$ is present since we need both the magnitude and phase of the FFT to recover the discrete signal. By trying out different values of $N_{trunc}$ on various sample spiral images, we find that $N_{trunc}=150$ gives a good compromise between the accuracy of the discrete signal and the number of parameters required to represent it.

To illustrate this in our running example, N=1000 and $N_{coeff}=501$, we first truncate the FFT (both magnitude and phase) with $N_{trunc}=150$ coefficients. Then, we take the inverse FFT [17] of the *truncated* FFT to get the *approximate discrete signal*. Fig. 3a shows the original discrete signal, overlayed by the approximate discrete signal, which requires 2×150=300 coefficients. The RMS error between the two signals is 3.80 %. The figure shows that the match between the two signals is visually very good, and we can effectively replace the original with the approximate discrete signal, which is a factor of $\frac{N_{original-pixels}}{300}=\frac{3228}{300}=10.76$ reduction in the number of parameters.

### 3.3 Distorted archimedes spiral

**Fig. 4:**
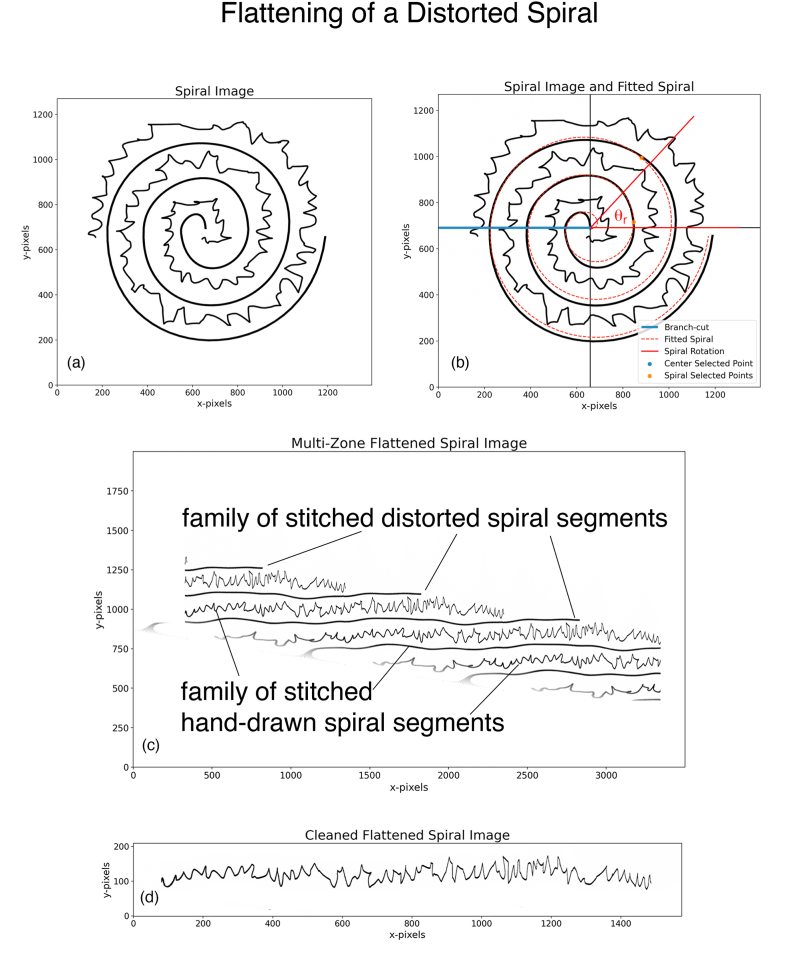
(a) Image in *Spiral Space* showing the distorted spiral and the hand-drawn spiral. **(b)***Spiral Space* of a and the fitted spiral. **(c)** Flattened Image of (a). **(d)** Cleaned stitched hand drawing (in Photoshop) from the image in (c).

In this paper so far, we have dealt with a perfect Archimedes spiral defined by (1). However, in practice, many hospitals and clinics routinely analyze patients' hand-drawn spirals by using a non-ideal distorted spiral. An example of this spiral used in actual practice is shown in Fig. 4a. The patient's hand drawing is simulated by us, but the spiral itself is a scan of an actual spiral used in practice. A cursory glance shows that it is not a perfect Archimedes spiral. We show in this section that by simply adding a new step our approach also works well on this distorted spiral. All the steps in the above two sections remain unchanged, we simply insert a new step between steps 1–3 and 1–4.

#### 3.3.1 Results of steps 1-1 through 1–3 of the above sections

We begin with the distorted spiral image of Fig. 4a. After selecting the center and two points on this distorted spiral, we obtain an equation for the Archimedes spiral to fit the distorted spiral. The result is shown in Fig. 4b. The fitted Archimedes spiral does not completely follow the distorted spiral. Flattening Fig. 4a by using the equation of the fitted Archimedes spiral gives us the family of flattened spiral images shown in Fig. 4c. Since the fitted spiral in Fig. 4b does not exactly follow the distorted spiral, all the stitched spirals have a low amplitude, slowly varying wave superimposed on them. For example, the distorted spiral does not map into a family of horizontal straight lines.

#### 3.3.2 Step 1–3.5 manually clean the flattened image

The superimposed waves make it difficult to select a rectangle to cleanly crop out a single stitched patient's spiral since the rectangle would also include pixels from other parts of the image. This additional step is to clean the image of Fig. 4c in an image editing application, such as Photoshop, and erase the unwanted pixels in the cropped region. The result is shown in Fig. 4d. The rest of the steps can be followed without modification. It is important to keep the x = 0 edge in the cropped region so that our program can calculate the $N_{original-pixels}$ accurately. We resample the signal to N=2000 samples in this example.

#### 3.3.3 Results of steps 1–4 through 1–5 of the above sections

**Fig. 5:**
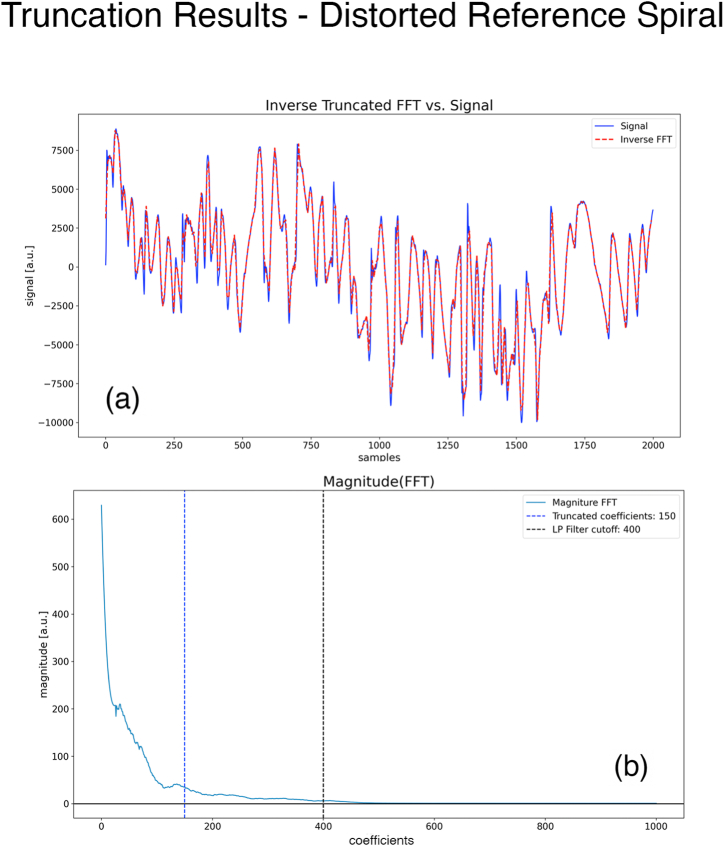
(a) Discrete signal obtained from Fig. 4d, overlaid by the approximate discrete signal obtained from the FFT shown in (b) truncated to 150 coefficients. The RMS error between the two signals is 10.06 % **(b)** FFT of the discrete signal in (a). The dashed blue line marks the 150 coefficients.

By following these steps, we obtain the normalized discrete signal with 2000 samples as shown in Fig. 5a.

#### 3.3.4 Steps 2-1 through 2–3 of the above sections

We determine the real FFT of the discrete signal (with $\;\frac{N}{2}+1=1001\;\text{coefficients})$ and truncate the FFT by $N_{trunc}=150$. The truncation value is marked in Fig. 5b by a blue dashed line. The final approximate discrete signal is obtained via the inverse FFT of the cropped FFT. Fig. 5a shows the original discrete signal with 2000 samples overlayed by this approximate discrete signal. The RMS error between the two signals is 10.06 %. We observe that even for a distorted spiral, the visual match between the two signals is very good, and we can replace the original with the approximate discrete signal requiring only 2×150=300 coefficients for most applications.

### 3.4 Cohort of hand-drawn spirals

To demonstrate the applicability and reproducibility of our procedure we used a total of 18 hand-drawn. We asked volunteers to simulate the hand-drawn spirals to represent mild to moderate tremors of varying amplitudes and frequencies. These spirals have been uploaded to our public GitHub repository [21] where they have been labeled from S1 to S18. A table of relevant parameters for each spiral has also been placed in the repository. Fig. 1, Fig. 2, Fig. 3 use spiral S2. The distorted spiral S3 is used in Fig. 4. Spiral S3 was obtained by Xeroxing a paper document that contained the reference distorted spiral and the spiral was hand-drawn on the scanned image by using a digitizing pen. The contrast was automatically adjusted to remove the light grey areas resulting from the Xeroxing process. For the rest of the spirals, the reference spiral with a given angle of rotation was programmatically drawn to produce a digital image. A digital pen was then used to hand-draw the spiral. We made sure that the spiral images contained an assortment of artifacts that make extracting the signal challenging, such as reference spirals having an unspecified angle of rotations, and hand-drawn lines with multiple pixel widths composed of varying grey scale values. Some samples also contain hand-drawn lines crossing the reference spirals or having gaps and loops. Additionally, some hand-drawn spirals have detectable tremor peaks in their spectra. This is summarized in a table in the repository.

Our procedure was applied to all 18 spiral images. The RMS error of the difference between the untruncated discrete signals and the inverse transform of the truncated (to 150 coefficients) FFTs was evaluated for the 18 samples. We obtain a mean RMS error of 6.26 % and a standard deviation of this RMS error of 3.22 % across the 18-sample cohort.

### 3.5 Time-dependent information and tremor frequency

**Fig. 6:**
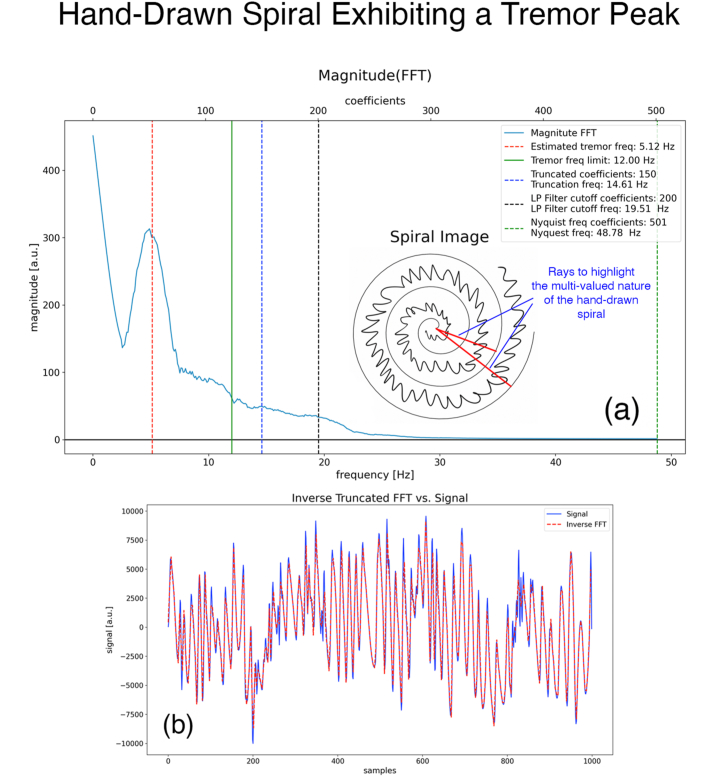
(a) Magnitude FFT obtained from the spiral shown in the inset. The spiral has the same pixel dimensions as the spiral in Fig. 2 (d) **(b)** Discrete signal obtained from the spiral shown in the inset of (a), overlaid by the approximate discrete signal obtained from the FFT shown in (a) truncated to 150 coefficients. The dashed blue line in (a) marks the 150 coefficients. The RMS error between the two signals is 12.67 %.

A strength of our procedure is that it can potentially be used to determine the tremor frequency from the magnitude FFT of the discrete signal (obtained from the flattened image of the hand-drawn spiral). Since frequency is inherently defined in terms of time, a spatially drawn spiral in itself cannot give us frequency information unless the time required to draw the spiral, $t_{to-draw}$, is also recorded. Given this additional information, the horizontal scale of the FFT can be expressed in terms of Hz (in place of coefficients). We have recorded $t_{to-draw}$ times for most of our samples and uploaded them to our GitHub public repository [21]. An example magnitude FFT of a sample that shows a tremor frequency peak is shown in Fig. 6a.

We estimate the frequency of the tremor to be 5.12 Hz, red dashed line, by roughly counting the peaks of the “wiggles” in the hand-drawn spiral (shown in the inset of the figure). We observe that the peak aligns nicely with the estimated tremor frequency. For this example, $t_{to-draw}=11.7\;\sec$. We see similar alignment of the peaks with estimated tremor frequencies in other samples which show peaks in their magnitude FFTs. Admittingly, counting the peaks is a crude method to estimate the tremor frequency. In our planned future formal study, we will use a motion capture camera system to measure the tremor frequency accurately.

### 3.6 Tremor amplitude

Besides the tremor frequency, our procedure also allows us to obtain a measure of the tremor amplitude by calculating the RMS value of the amplitude of the one-dimensional signal. The RMS values for our current 18-sample cohort have been evaluated and uploaded to our GitHub repository [21]. This RMS value is in pixel units and not actual limb amplitude units of the patient.

### 3.7 Time required to apply all the steps in the procedure

We also timed 10 complete runs of our procedure from start to finish and obtained a mean time of 1 min 56 s and a standard deviation of 3.13 s. This timing was done on a laptop manufactured in the year 2000 (Mac Book Pro). However, if the reference Archimedes spiral is distorted, or if the hand-drawn spiral crosses the reference spiral, we need an additional step to clean up the flattened image, as discussed earlier. In our experience this step can add about 2 min in Photoshop (for the spiral discussed previously), or longer if elaborate editing is required. The timing results can be found in our repository [21].

## 4 Discussion

This paper has addressed the problem of automatically extracting single-valued one-dimensional signals directly from images containing hand-drawn spirals obtained either from Xeroxed and scanned paper documents or from digital tablets. In methods proposed in existing literature[[9], [10], [11], [12], [13], [14], [15]], the spirals are hand-drawn on a digitizing tablet, and features for machine learning models are extracted from this data. We have shown that our alternate approach has the advantage of giving us an interpretable one-dimensional mathematical signal directly from a hand-drawn image. This enables us to use efficient signal processing algorithms such as the FFT to transform the derived signal and calculate important quantities like truncation errors and tremor frequencies. Furthermore, our approach also has the potential to use the truncated set of parameters as a feature set for machine learning models. We have also demonstrated the feasibility and robustness of our approach on a cohort of 18 hand-drawn spiral images[21] containing a range of challenging artifacts and obtained a mean RMS truncation error of 6.26% and a standard deviation of the RMS of 3.22% across the cohort. We have also shown that by timing the process of drawing the spirals we can label the frequency axis in units of Hz (instead of coefficients), which provides us further intuition about assessing the truncation of parameters used in our procedure. For PD (Parkinson’s Disease) and ET (Essential Tremor), we are interested in a tremor frequency no larger than 12 Hz, indicated by the solid green line in Fig. 6(a). As long as the truncation frequency corresponding to the 150 coefficients (blue dashed line) lies near or higher than the green line, our truncated signal will preserve the important features relevant to the physical characteristics of the tremors we are interested in. We certainly would like the truncation frequency to be higher than the frequency of the tremor (red dashed line). We note that these conditions are met in all our 18 samples. The black dashed line shows the cutoff of the LP (Low Pass) filter in Hz used to eliminate the high-frequency noise in the data. As expected, this frequency lies higher than all the other relevant frequencies. The untruncated discrete signal (solid blue) is plotted against the truncated discrete signal (dashed red) for this sample in Fig. 6(b). The RMS error of truncation for this signal is evaluated to be 12.67%. Even though this error appears to be relatively high, we note that according to Fig. 6(a) the frequencies of interest below 12 Hz (especially the tremor peak at 5.12 Hz), are preserved since they lie below the truncation frequency of 14.61 Hz. The RMS error arises from frequencies higher than 14.61 Hz. This is reflected in Fig. 6(b), in which the two curves follow each other very closely over the majority of the time except for the very small intervals where the hand-drawn spiral exhibits very sharp edges. Note that the hand-drawing is multiple-valued (please see the section below on limitations of our method), however, this has not affected the frequency peak in the spectrum. Finally, a strength of our method is that it could be used to determine the tremor frequency even if the tremor amplitude is extremely weak.

## 5 Limitations of the method

At the beginning of this paper, we listed various artifacts present in Xeroxed/scanned documents and digital tablet files that make it challenging to extract a clean signal from the digital image. Throughout this paper, we have shown that our procedure works well in extracting the signal in the presence of these artifacts. However, our methodology does have its limits, and there is potential for future improvements. We now elaborate on these limitations and comment on potential improvements and fixes: i) The hand-drawn spiral could intersect the reference Archimedes spiral. We could handle this situation similar to the problem associated with distorted spirals discussed earlier and edit the image in an image-editing software. A superior and more efficient solution would be to make the patient draw the spiral in a color different than the reference spiral, then our software could be made to leverage this difference and the time-consuming image editing step could be avoided. ii) Another problem can be posed by dark marks in the image caused by Xeroxing or scanning. If the offending marks are lighter than the patients’ spiral (which is almost always the case), then our current algorithm would continue to work since it looks for pixels with strong enough densities to determine the signal. Our technique works even better if the contrast of the original image is enhanced before processing. Extremely strong marks could be removed in an image editing software. Better still, if the patient draws in a non-grey saturated color, i.e., a saturated red or cyan, then we could easily detect and ignore these marks in our algorithm since scanning marks tend to be of non-saturated color. iii) An additional problem can be caused by gaps in the hand-drawn spiral which will cause our discrete signal to jump to a high value during the gaps. As a fix, our algorithm could detect these values during a pre-processing step and replace the offending values in the signal with NaN values. Next, we could use an available Python package like pandas.DataFrame.interpolate [26], which is capable of replacing NaN values by interpolating between the edges of the missing data in the gaps. iv) As illustrated in Fig. 6(a - inset), the drawing could potentially have closed loops or slanted structures that cause a ray (e.g., red rays in the figure) to intersect the hand-drawn spiral at more than one point, causing the flattened drawing to become multiple-valued. When our algorithm determines the discrete signal, for a given x pixel on the flattened image, it starts looking for y pixel values of sufficient strength (in increasing y-values) and stops as soon as it detects the first value. Thereby it latches on to the lower portion of the curve and ignores the upper part of the multi-valued curve. This does not have a decremental effect on determining the tremor frequency (please see the next subsection), but it does cause our algorithm to underestimate the rms value of the non-normalized discrete signal, depending on the density of multivalued pixels in the drawing. We plan on incorporating these modifications into our algorithm in the future and also updating our GitHub repository. Our procedure becomes more time-consuming as the severity of the tremor increases because of the additional step required to clean up the flattened image. Therefore, our method is suitable for early-stage to moderate tremors. Specifically, our method cannot be used when the tremor is so strong that the hand-drawn spiral ceases to resemble the reference spiral. Ideally, a production quality modification of our program would incorporate a Graphical User Interface (GUI) with inbuilt image editing facilities, which would eliminate the time required for switching to an external image editor.

## 6 Future investigations

We also plan on using the small feature sets (obtained from the patients) consisting of the relatively few coefficients to train classification ML (Machine Learning) models to make predictions about the tremors. We show in this paper that a *one-dimensional* signal extracted from the hand-drawn spiral can be reconstructed by as few as 300 coefficients (150 from the magnitude FFT and 150 from the phase FFT), even though the signal itself could be composed of thousands of samples, $N_{original-pixels}=3228$ in our running example which leads to about a 10-fold reduction in parameters. If the goal is to extract a feature set for ML, we may need only the magnitude coefficients which reduces the feature set to about 150 (plus 1 feature representing the RMS value). This reduction of features, from thousands to a few hundred, can have important implications for applications that leverage machine learning to make predictions from hand-drawn spirals. For an ML prediction to generalize well in the real world, the amount of data (i.e., the number of hand-drawn spirals) for training should be larger than the number of features [27], otherwise, there is a real possibility of overfitting [27] and poor generalizability. Since we typically don't have access to thousands of hand-drawn spirals, this is a notable limitation on using machine learning for spiral data. Using thousands of signal samples as features would potentially require thousands of hand-drawn samples; however, if we use only 300 (or 150) features, then machine learning becomes a viable option. We note that the coefficients described in this paper are derived from a normalized signal, hence information about the actual amplitude of the tremor has been lost. We would therefore need to add the RMS value of the discrete signal before it is normalized to the total feature set, which will increase the feature set by only one. The RMS values for our current 18-sample cohort have been evaluated and uploaded to our GitHub repository [21]. This RMS value is in pixel units and not actual limb amplitude units of the patient.

## 7 Conclusions

We have devised a semi-automatic procedure requiring minimal user intervention to automatically extract a patient's hand-drawn spiral as a discrete *one-dimensional* signal from a scanned image or a drawing on a digital tablet's screen. Our procedure overcomes the problem that spirals in an image are of multi-pixel width with varying pixel greyscale (or color) values. Furthermore, our procedure does not require precise placement of the paper (with the original image) on the scanner's bed, it can be placed anywhere and at any angle. The fitted spiral, determined from the two points selected by the user, assesses the angle and scale of the spiral. Once the discrete *one-dimensional* signal is extracted, it can be analyzed mathematically to address problems in various neurological domains. This study also shows that our procedure works well even for distorted spirals used by some for spiral analysis. Some hand editing in an image editing program is required for distorted spirals or overly complex hand drawings. Finally, we show the limitations and feasibility of using our method to determine the tremor frequency of PD or ET patients.

### Declaration of generative AI and AI assisted technologies in the writing process

All writing was performed by the named authors; AI was not used for the writing of this paper.

## Financial disclosure/conflict of interest

All the authors have no relevant financial, non-financial, or conflict of interest to disclose for the research related to this manuscript.

## Funding sources

No grants, financial support, gifts, or honoraria were paid by any agency or company for the research related to this manuscript.

## Data availability statement

All the data, user guide, and software developed for the project in this paper have been made available in the authors’ public GitHub repository at https://github.com/khanosu/Spiral-Paper-1.git under the open-source GNU General Public License [21].

## CRediT authorship contribution statement

**Furrukh Khan:** Writing – original draft, Methodology, Investigation, Formal analysis, Conceptualization. **Jessie Xiaoxi:** Software, Investigation, Conceptualization. **Andrew Uehlin:** Writing – review & editing, Validation. **Brian Dalm:** Writing – review & editing, Investigation, Conceptualization. **Evan Thomas:** Writing – review & editing, Project administration, Investigation, Conceptualization.

## Declaration of competing interest

The authors declare that they have no known competing financial interests or personal relationships that could have appeared to influence the work reported in this paper.
